# Highly Efficient Phosphorescent Blue-Emitting [3+2+1] Coordinated Iridium (III) Complex for OLED Application

**DOI:** 10.3389/fchem.2021.758357

**Published:** 2021-10-07

**Authors:** Zijian Liu, Si-Wei Zhang, Meng Zhang, Chengcheng Wu, Wansi Li, Yuan Wu, Chen Yang, Feiyu Kang, Hong Meng, Guodan Wei

**Affiliations:** ^1^ Tsinghua-Berkeley Shenzhen Institute (TBSI), Tsinghua University, Shenzhen, China; ^2^ PURI Materials, Shenzhen, China; ^3^ Institute of Materials Research, Tsinghua Shenzhen International Graduate School, Tsinghua University, Shenzhen, China; ^4^ Peking University Shenzhen Graduate School, Peking University, Shenzhen, China

**Keywords:** OLED, Iridium (III) complexes, blue emitters, transient absorption, quantum yield

## Abstract

Cyclometalated iridium (III) complexes are indispensable in the field of phosphorescent organic light-emitting diodes (PhOLEDs), while the improvement of blue iridium (III) complexes is as yet limited and challenging. More diversified blue emitters are needed to break through the bottleneck of the industry. Hence, a novel [3+2+1] coordinated iridium (III) complex (noted as **Ir-dfpMepy-CN**) bearing tridentate bis-N-heterocyclic carbene (NHC) chelate (2,6-bisimidazolylidene benzene), bidentate chelates 2-(2,4-difluorophenyl)-4-methylpyridine (dfpMepy), and monodentate ligand (-CN) has been designed and synthesized. The tridentate bis-NHC ligand enhances molecular stability by forming strong bonds with the center iridium atom. The electron-withdrawing groups in the bidentate ligand (dfpMepy) and monodentate ligand (-CN) ameliorate the stability of the HOMO levels. **Ir-dfpMepy-CN** shows photoluminescence peaks of 440 and 466 nm with a high quantum efficiency of 84 ± 5%. Additionally, the HATCN (10 nm)/TAPC (40 nm)/TcTa (10 nm)/10 wt% **Ir-dfpMepy-CN** in DPEPO (10 nm)/TmPyPB (40 nm)/Liq (2.5 nm)/Al (100 nm) OLED device employing the complex shows a CIE coordinate of (0.16, 0.17), reaching a deeper blue emission. The high quantum efficiency is attributed to rapid singlet to triplet charge transfer transition of 0.9–1.2 ps. The successful synthesis of **Ir-dfpMepy-CN** has opened a new window to develop advanced blue emitters and dopant alternatives for future efficient blue PhOLEDs.

## Introduction

Cyclometalated iridium (III) complexes have attracted enormous attention in the field of phosphorescent organic light-emitting diodes (PhOLEDs) due to their relatively short triplet lifetime and high phosphorescence quantum yields (Geffroy et al., 2006). However, the stability of organic luminescence materials at high temperature are worse than inorganic materials, especially the inorganic upconversion materials (Wang et al., 2021a; Wang et al., 2021b). Besides, compared with red and green iridium (III) complexes, the development of blue iridium (III) complexes for PhOLEDs is still limited and challenging (Brown et al., 2004). Several proven strategies benefit the achievement of blue emitters: (Ma et al., 2020). 1), stabilizing the highest occupied molecular orbital (HOMO) by introducing electron-withdrawing groups on the phenyl ring, like perfluoro carbonyl (Lee et al., 2013), pentafluorophenyl (Tsuzuki et al., 2003), carborane (Furue et al., 2016), sulfonyl (Lim et al., 2017; Lin et al., 2014), dimesitylboron (Lorente et al., 2017), or formyl (Bin Mohd Yusoff et al., 2017). 2), elevating the lowest unoccupied molecular orbital (LUMO) by grafting electron-donating groups onto pyridine moieties, like methoxy group on the pyridyl ring (Yam and Lo, 2006). These two strategies positively influence the energy gap of the iridium (III) emitters, which promote the hypsochromic photoluminescence spectrum. For achieving high phosphorescence quantum yields, a few essential factors should be taken into account, among which the most significant one is to enhance the contribution of the metal to ligand charge transfer (MLCT) in the triplet manifold. The metal d_π_ orbital’s involvement increases the coupling of the orbital angular momentum to the electron spin, which enhances the spin-orbit coupling term and drastically decreases the radiative lifetime, and hence realizes the possibility of achieving high quantum yield (Li et al., 2005; Wilkinson et al., 2006).

**SCHEME 1:**
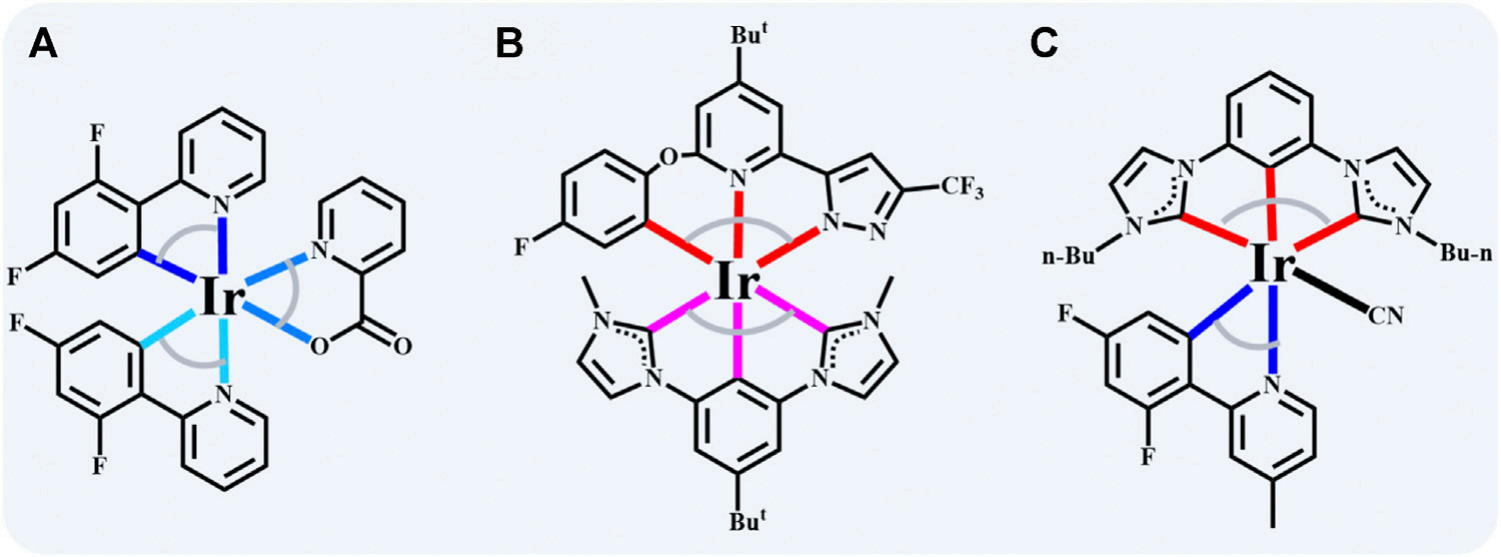
The typic blue emitters of iridium (III) complexes with **(A)** [2+2+2] (**FIrpic**) (Adachi et al., 2001), **(B)** [3+3] (**Ir(minb)(pzpy** ^**Bu**^ **Oph** ^**F**^ **)**) (Kuo et al., 2017) and **(C)** [3+2+1] (**Ir-dfpMepy-CN** in this work) conformation.

The blue iridium (III) complexes generally feature an octahedral conformation with three bidentate or two tridentate ligands (labeled as [2+2+2] and [3+3], respectively) due to the *d* ^*6*^ electron configuration of iridium ion (Bin Mohd Yusoff et al., 2017). [2+2+2] Coordinated iridium (III) complexes’ inherent superior performance benefits from the diversity of bidentate chelates (Adachi et al., 2001; Holmes et al., 2003), and one of the most typical representatives is greenish-blue bis (4’,6’-difluorophenylpyridinato) iridium (III) picolinate (FIrpic) (Adachi et al., 2001). As shown in Scheme 1A, incorporating the two strong electron-withdrawing groups (dfppy) facilitates the enlargement of the bandgap of iridium (III) complexes by stabilizing the HOMO levels (Holmes et al., 2003) [3+3]. Coordinated iridium (III) complexes show excellence in rigidity and durability due to the stronger metal-ligand bonding interaction, making the d–d excited states or other unspecified quenching states destabilized to enhance the stability of the complexes (Kuo et al., 2017; Kuo et al., 2018; Gnanasekaran et al., 2019; Hsu et al., 2019). Scheme 1B demonstrates a typical [3+3] counterpart, and the interrupted conjugation changes the nature of the frontier orbitals, achieving a blue emission of CIE (0.15, 0.17) (Kuo et al., 2017). Multi-strategies should be utilized together to acquire high-efficiency blue light emitters. Even though some iridium (III) complexes present plausible molecular properties, blue emission severely restricts material selection for PhOLEDs. Consequently, it is imperative to extend the existing iridium (III) complexes system to obtain blue PhOLEDs with high color purity and efficiency.

In this work, a novel [3+2+1] coordinated iridium (III) complex, bearing tridentate bis-N-heterocyclic carbene (NHC) chelate (2,6-bisimidazolylidene benzene), bidentate chelates 2-(2,4-difluorophenyl)-4-methylpyridine (dfpMepy), and monodentate ligand (-CN), has been designed and synthesized. The tridentate ligand improves the molecular stability with the similar function of the [3+3] conformation and sufficient bidentate ligands are available to tune the emission wavelength. Additionally, monodentate ligand (-CN) is a strong electron-withdrawing group, facilitating the stability of the HOMO levels. The designed [3+2+1] iridium (III) complex shows emission peaks of 440 and 466 nm with a high quantum efficiency of 84 ± 5%. Furthermore, the femtosecond transient absorption spectrum further reveals the singlet transition to triplet state with a time constant of 0.9–1.2 ps. This work exhibits a novel cyclometalated iridium (III) complex configuration that will help develop more dopant options for blue PHOLEDs.

## Materials and Methods

### Synthesis of 2-(2,4-Difluorophenyl)-4-Methylpyridine (dfpMepy)

2-bromo-4-methylpyridine (6.84 g, 40 mmol), potassium carbonate (8.29 g, 60 mmol) and tetrakis (triphenylphosphine) palladium (1.49 g, 1.2 mmol) were added into a 250 ml round-bottomed flask. (2,4-Difluorophenyl) boronic acid (4.67 ml, 40 mmol), ethanol (20 ml) and tetrahydrofuran (20 ml) were slowly added into the flask under stirring conditions. The mixture was then heated to reflux for 20 h under a nitrogen atmosphere. After cooled to room temperature, the resulting solution was firstly washed by water for three times and then filtered, extracted with dichloromethane, and dried with MgSO_4_, yielding an oily organic layer. The crude product was concentrated and purified by column chromatography (with eluent hexane/DCM, v/v = 5/1) to obtain light yellow powder (6.39 g, 78%). ^1^H NMR (400 MHz, Chloroform-d): *δ* = 8.58 (d, *J* = 5.0 Hz, ^1^H), 7.98 (td, *J* = 8.9, 6.7 Hz, ^1^H), 7.58 (s, ^1^H), 7.13–7.07 (m, ^1^H), 7.01 (td, *J* = 8.3, 2.6 Hz, ^1^H), 6.93 (ddd, *J* = 11.3, 8.8, 2.6 Hz, ^1^H), 2.43 (s, ^3^H) ppm.

### Synthesis of 1,3-Bis(imidazolyl)Benzene (1)

1,3-Dibromobenzene (5 ml, 42 mmol), imidazole (7 g, 104 mmol), potassium carbonate (14.4 g, 104 mmol), cupric oxide (0.83 g, 10.4 mmol) and DMSO (60 ml) were added into a 250 ml flask. The mixture was heated to 150°C for 48 h. After cooling to room temperature, the resulting solution was filtered under reduced pressure and washed by water for three times. DMSO was then removed from the solution by vacuum distillation. The crude product was concentrated and purified by column chromatography to obtain a white powder (5.8 g, 66%). ^1^H NMR (400 MHz, Chloroform-d): *δ* = 7.93 (s, ^2^H), 7.63 (t, *J* = 8.0 Hz, ^1^H), 7.45 (s, ^2^H), 7.42 (d, *J* = 1.9 Hz, ^1^H), 7.35 (s, ^2^H), 7.27 (s, ^2^H) ppm.

### Synthesis of 1,1’-(1,3-Phenylene) bis(3-Butyl-1H-Imidazolium) Bromide (2)

1,3-Bis(imidazolyl)benzene (2.9 g, 13.8 mmol), n-butyl bromide (9.5 g, 69 mmol) and acetonitrile (30 ml) were added to a 100 ml round-bottomed flask. The mixture was heated to 85°C for 3 h. After cooled to room temperature, the solution was concentrated and purified by vacuum distillation to obtain a white powder (9.5 g, 81%). ^1^H NMR (400 MHz, DMSO-d6): δ = 10.29 (s, ^2^H), 8.59 (t, *J* = 1.7 Hz, ^2^H), 8.53 (*t*, *J* = 1.9 Hz, ^1^H), 8.19–8.13 (m, ^2^H), 8.07 (dd, *J* = 8.2, 1.7 Hz, ^2^H), 7.96 (dd, *J* = 8.9, 7.8 Hz, ^1^H), 4.33 (*t*, *J* = 7.2 Hz, ^4^H), 1.93 (*p*, *J* = 7.4 Hz, ^4^H), 1.36 (h, *J* = 7.8, 7.3 Hz, ^4^H), 0.95 (*t*, *J* = 7.4 Hz, ^6^H) ppm.

### Synthesis of Ir-dfpMepy-Br

[Ir(COD)Cl]_2_ (500 mg, 0.745 mmol) and compound 2 (475 mg, 1.118 mmol) were added into a 25 ml Schlenk tube. Triethylamine (2 ml) and acetonitrile (10 ml) were slowly added into the Schlenk tube under the protection of N_2_. The mixture was heated to 90°C for 12 h. After cooled to room temperature, the resulting solution was concentrated by vacuum distillation to get a yellow sticky solid. Then dfpMepy (230 mg, 1.118 mmol) and propionic acid (10 ml) were added for next step reaction with the residual. The mixture was heated to 150°C under N_2_ atmosphere for 24 h. After cooled to room temperature, the resulting solution was purified by vacuum distillation, column chromatography to get crude product Ir-dfpMepy-Br (320 mg, 58%). ^1^H NMR (400 MHz, DMSO-d6): δ = 10.13 (d, *J* = 5.8 Hz, ^1^H), 8.16 (s, ^1^H), 8.04 (d, *J* = 2.0 Hz, ^2^H), 7.47 (d, *J* = 5.9 Hz, ^1^H), 7.40 (d, *J* = 7.8 Hz, ^2^H), 7.23 (d, *J* = 2.0 Hz, ^2^H), 7.18 (t, *J* = 7.7 Hz, ^1^H), 6.50 (t, *J* = 11.4 Hz, ^1^H), 5.27 (dd, *J* = 9.0, 2.5 Hz, ^1^H), 2.59 (s, ^2^H), 1.36–1.27 (m, ^2^H), 0.95 (s, ^2^H), 0.81 (td, *J* = 11.8, 10.5, 6.9 Hz, ^2^H), 0.65 (d, *J* = 3.7 Hz, ^6^H) ppm.

### Synthesis of Ir-dfpMepy-CN

Ir-dfpMepy-Br (250 mg, 0.337 mmol) and Silver cyanide (67.68 mg, 0.506 mmol) were dissolved in N. N-dimethylformamide (10 ml) in Schlenk tube. The mixture was then heated to 120°C under N_2_ atmosphere. After cooled to room temperature, the resulting solution was further filtered through kieselguhr, evaporated under reduced pressure and purified by silica gel column to get a white powder (206.71 mg, 89%). ^1^H NMR (400 MHz, Chloroform-d): *δ* = 9.98 (s, ^1^H), 8.24 (s, ^1^H), 7.50 (s, ^2^H), 7.26 (s, ^1^H), 7.16 (d, *J* = 7.5 Hz, ^3^H), 6.80 (s, ^2^H), 6.32–6.22 (m, ^1^H), 5.49 (s, ^1^H), 3.27 (d, *J* = 14.5 Hz, ^4^H), 2.61 (s, ^3^H), 1.40 (s, ^2^H), 1.15 (d, *J* = 3.7 Hz, ^2^H), 0.94 (s, ^2^H), 0.80 (s, ^2^H), 0.77 (s, ^6^H) ppm.

### Materials Characterization

All the reagents were purchased from Leyan, General Reagent and Aldrich and all the reactants and solvents were used without further purification unless otherwise specified. The key reaction products are characterized by a 400 MHz ^1^H-NMR spectrometer (Bruker AV400) and referenced to tetramethylsilane (TMS) as a standard benchmark at 0.00 ppm. The ultra-violate absorption spectra was performed by Cary 5000 UV-vis-NIR spectrophotometer in degassed dichloromethane at a diluted concentration (2 × 10^−5^ M) of the iridium (III) complex. The photoluminescence (PL) spectra was obtained by spectrofluorometer of Edinburgh Instruments Ltd FS5. The femtosecond transient absorption (fs-TA) measurements were obtained on a Helios pump-probe system (Ultrafast Systems LLC) with an amplified femtosecond laser system (Coherent 35 fs, 1 kHz, 800 nm). The 320 nm pump pulses were achieved by the optical parametric amplifier (TOPAS-800-fs). The 380–680 nm probe pulses were gained by focusing the tiny portion of the 800 nm laser beams onto a sapphire plate. The fs-TA curves were collected and further analyzed by Surface Xplorer software. Thermogravimetric analysis (TGA) of **Ir-dfpMepy-CN** was recorded by the Mettler TGA2 thermogravimeter. The weight loss of the iridium complex was initially measured from 25 to 100°C at a speed of 10°C/min under a nitrogen atmosphere. Before continuing to heat to 500°C, the sample was kept for 15 min at 100°C. Electrochemical characterization of the iridium complex was measured by PalmSens4 electrochemical workstation, using platinum wire as the counter electrode, platinum-carbon as a working electrode and a saturated calomel electrode (SCE) in a saturated KCl aqueous solution as the reference electrode. The cyclic voltammogram of **Ir-dfpMepy-CN** was referenced to the ferrocene/ferrocenium couple at the scanning rate of 100 mV s^−1^.

### Device Fabrication

The indium-tin-oxide (ITO) coated glass substrates were initially cleaned sequentially by deionized water, ethanol, dichloromethane for 5 min separately and then treated with plasma for 1 min. The dopant material **Ir-dfpMepy-CN** and DPEPO were thermally deposited together at the speed of 0.1 Å s^−1^ and 0.9 Å s^−1^ separately, while the other organic functional layers were evaporated at the same rate of 1.0 Å s^−1^ at a pressure of ca. 3.5 × 10^–7^ Torr. A 2.5 nm Liq layer was deposited on Al cathode and the two materials are deposited at the rate of 0.1 and 1 Å s^−1^. The organic diode was assembled on an active area of 3 × 3 mm^2^ on the substrates. The device performance, including electroluminescence (EL) spectra, Commission Internationale de L’Eclairage (CIE) and current density-voltage-luminance (J-V-L) curves, were measured by a Keithley 2400 semiconductor characterization system with PR-788 photometer and BM-7A luminance colorimeter.

### Theoretical Calculation

The structures were optimized with dispersion density functional theory at the PBE0-D3/def2-SVP level by Gaussian 09. In order to investigate the photophysical properties, the excited electronic structures of these molecules were calculated at the PBE0-D3/def2-TZVP level with the time-dependent density functional theory (TDDFT) method. The electron transition characterization was obtained by electron excitation analysis performed using Multiwfn program from the transition density matrix of TDDFT calculation.

## Results and Discussion

**FIGURE 1:**
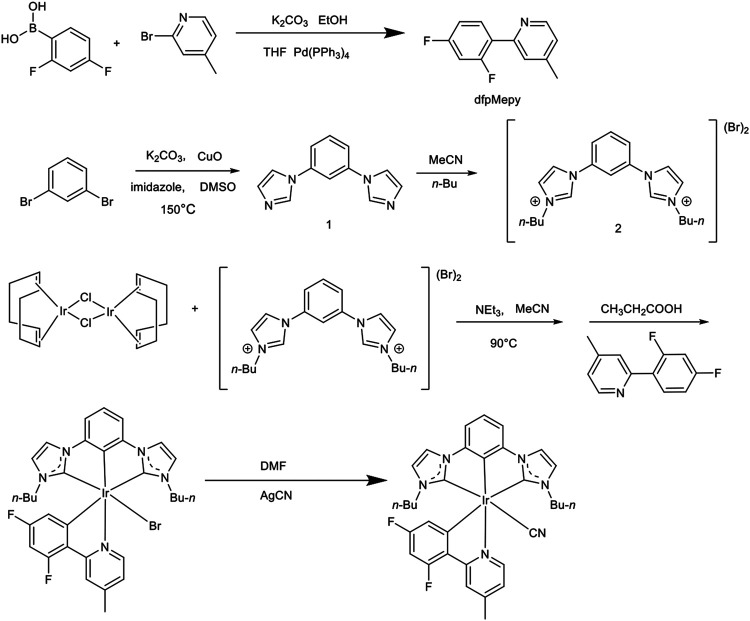
Synthetic route and chemical structure of the **Ir-dfpMepy-CN** with [3+2+1] conformation.

The synthetic route of the **Ir-dfpMepy-CN** with [3+2+1] conformation was depicted in Figure 1, and the detailed steps can be obtained in the supporting information (SI). In brief, the tridentate bis-N-heterocyclic carbene (NHC) chelate was synthesized by a two-step reaction of 1,3-dibromobenzene with imidazole and n-butyl bromide, yielding 66 and 81%, respectively. The bidentate ligand dfpMepy was directly synthesized by one step of the Suzuki coupling reaction in an alkaline condition. Controlling the equivalence ratio (1:1) of (2,4-difluorophenyl) boronic acid and 2-bromo-4-methylpyridine, the product was obtained in a relatively high yield of 78%. The -Br was substituted by the stronger electron-withdrawing group of cyanide (-CN), which could significantly influence the metal-centered molecular orbital due to its strong σ-donating p orbital and a low-lying π orbital (Hanusa, 2011). All the compounds were purified by vacuum distillation and column chromatography and subsequently characterized by ^1^H NMR spectrometry for further study, as shown in Supplementary Figures S1–S5. The tridentate bis-NHC chelate serves as electron-donating group in the molecule. Both the bidentate (dfpMepy) and monodentate (-CN) ligand are strong electron-withdrawing groups, expecting to enlarge the complex’s bandgap by destabilizing the LUMO and stabilizing the HOMO levels.

**FIGURE 2:**
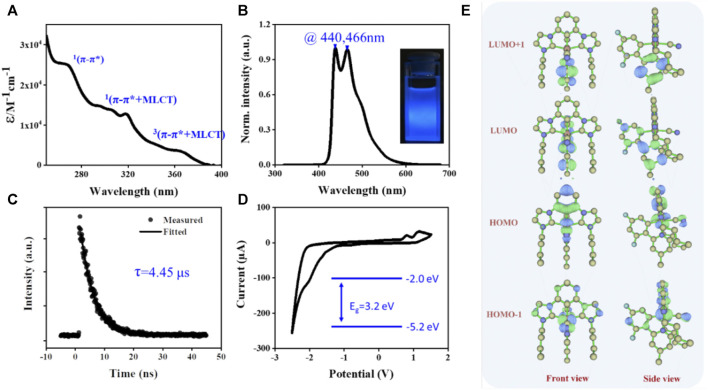
The photophysical properties of the [3+2+1] iridium (III) complex **Ir-dfpMepy-CN**. **(A)** the absorption spectra and **(B)** normalized PL spectra of diluted in CH_2_Cl_2_ solution. **(C)** Lifetime decay curves at 440 nm and **(D)** cyclic voltammograms (CV) in DCM with ^n^Bu_4_NPF_6_ (0.1 M) as supporting electrolytes. **(E)** The computed frontier molecular orbitals energy surfaces at the ground state (S_0_) optimized geometry.

The photophysical properties of the [3+2+1] iridium (III) complex were investigated. As shown in Figures 2A,B, the absorption and phosphorescent emission (PL) for the **Ir-dfpMepy-CN** were recorded in degassed CH_2_Cl_2_ (DCM) solution at a concentration of 2 × 10^−5^ M. The strong absorption at 250–280 nm in the ultraviolet region (ε > 2.5 × 104 M^−1^ cm^−1^) was assigned to the ligand-centered (^1^π→π*) transitions (Ashizawa et al., 2009). The absorption bands with small vibrational shoulders at 300–330 nm were ascribed to spin-allowed intra-ligand (^1^π→π*) transition and metal to ligand charge transfer (^1^MLCT) transitions. The weak absorptions bands (ε ≈ 0.3 × 104 M^−1^ cm^−1^) lying in the visible light region at 350–400 nm were related to the spin-orbit coupling enhanced ^3^π→π* states and spin-forbidden metal to ligand charge transfer (^3^MLCT) transitions (Chen et al., 2015). The PL spectra of **Ir-dfpMepy-CN** exhibited strong phosphorescent emission at around 430–470 nm with maximum peaks at 440 and 466 nm, which should be attributed to the ^3^MLCT ^3^LLCT, ^3^LC induced by spin-orbit coupling. The PL spectra of the designed **Ir-dfpMepy-CN** with [3+2+1] confirmation is even bluer than that of the classic blue-emitter FIrpic. More gratifyingly, the **Ir-dfpMepy-CN** showed a high absolute quantum efficiency of 84 ± 5%.

The corresponding lifetime decay curve of phosphorescence was presented in Figure 2C, revealing a lifetime of 4.6 μs. The electrochemical properties of the [3+2+1] iridium (III) complex in degassed DCM were assessed by cyclic voltammogram (CV). The curve showed positive oxidative peaks (E__ox_) of 0.80–1.16 V (vs. Fc^+^/0) due to the electron-deficient property of the pyridine group and reduction peak (E_red) around −2.50 V. The HOMO and LUMO energy of **Ir-dfpMepy-CN** were calculated by Eqs 1, 2 below, where the E_ox and E_red were obtained from the onset potential of the first oxidation and reduction peaks and the ferrocene redox value is −4.4 eV (Hack et al., 2005; Leonat et al., 2013). Calculated HOMO and LUMO of **Ir-dfpMepy-CN** is −5.2 and −2.0 eV. Thermogravimetric analysis (TGA) experiments revealed the thermal stability of **Ir-dfpMepy-CN** (Supplementary Figure S6). The complex shows good thermal stability, 2 wt% loss >270°C.

$$E_{\mathrm{HOMO}}=-\left(eE_{\mathrm{ox}}+\mathrm{4.4}\right)\left[\mathrm{eV}\right]$$ (1)

$$E_{\mathrm{LUMO}}=\left(\frac{\mathrm{1240}}{\lambda {\;}_{\mathrm{UV}}}+E_{\mathrm{HOMO}}\right)\left[\mathrm{eV}\right]$$ (2)

The density functional theory (DFT) and time-dependent density functional theory (TDDFT) calculation were implemented to gain a deeper insight into the ground and excited electronic states of the **Ir-dfpMepy-CN**. The two highest occupied and lowest unoccupied molecular orbitals (HOMO, HOMO-1, LUMO, LUMO+1) surfaces and their corresponding energy levels were given in Figure 2E. The LUMO is mainly located on the bidentate ligand (dfpMepy), and the HOMO is a mixed metal-ligand character with contributions from Ir 5d orbitals, tridentate bis- NHC chelate, and monodentate cyanide ligand. Specifically, the participation of Ir 5d orbitals in HOMO and HOMO-1 are 36.6 and 48.2%, and their energy levels are close (−5.70 and −5.78 eV). The large proportion of metals involved is the main reason for the high quantum efficiency of the **Ir-dfpMepy-CN**. The phenyl unit of bis-NHC pincer moiety is suggested to dominate the HOMO, while the difluoro-phenyl unit of dfppy dominates a major contribution in its HOMO-1. The participation of the bis-NHC carbene pincer moiety and monodentate ancillaries in HOMOs keep synergistically. The N-heterocyclic phenyl unit in dfpMepy almost dominates LUMO and LUMO+1. The T_1_ optical transition state were calculated by TD-DFT. As shown in Supplementary Table S1, the T_1_ transition state is contributed by HOMO-3, HOMO-2, HOMO-1 and LUMO. Supplementary Figure S8 shows the frontier molecular orbitals calculated at T_1_ geometries, indicating the T_1_ transition state is mixing MLCT, ligand centered (LC) or intra-ligand charge transfer (ILCT) excited state.

**FIGURE 3:**
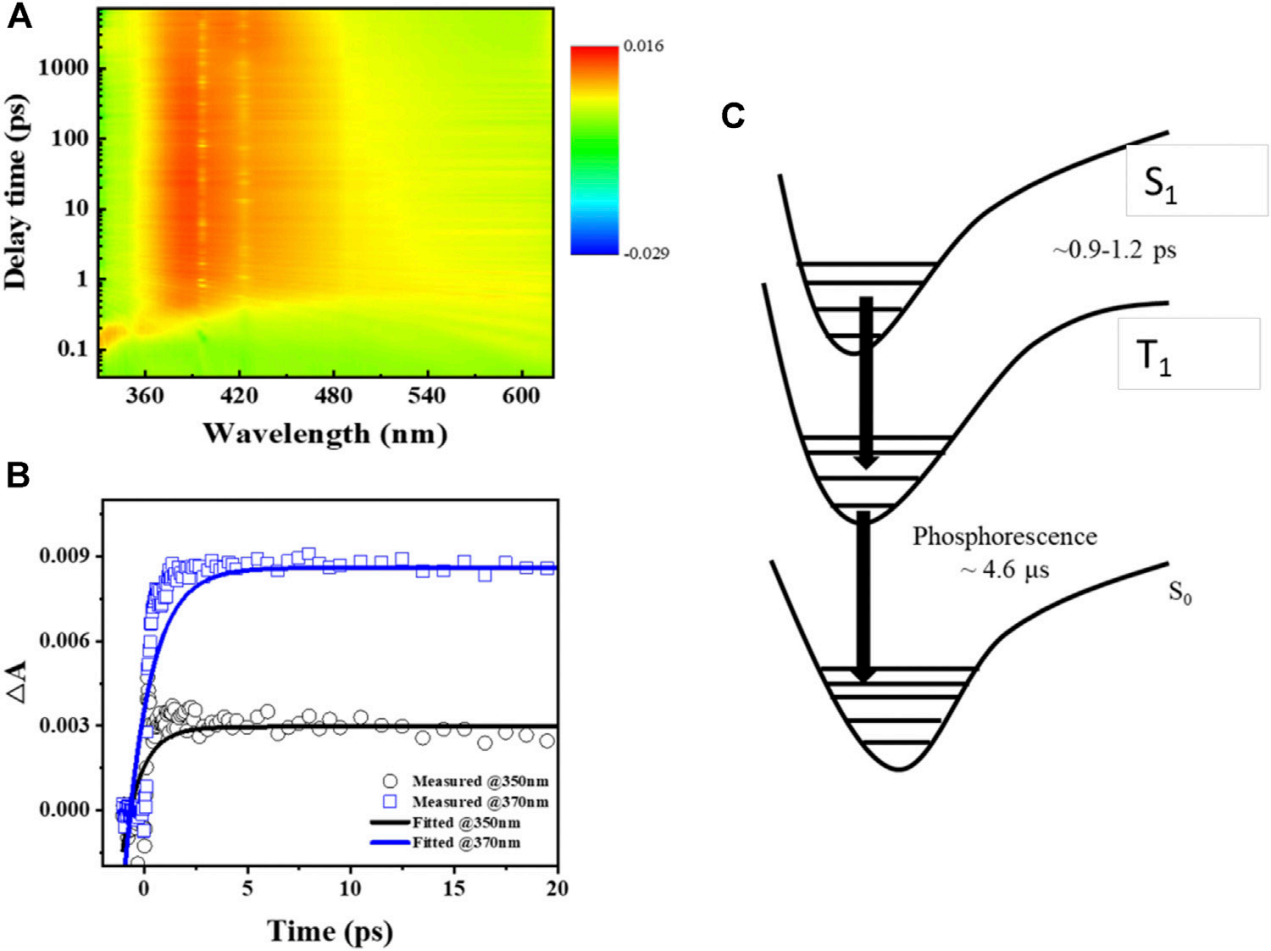
**(A)** Color plot of femtosecond transient absorption spectra of **Ir-dfpMepy-CN** in DCM at room temperature, **(B)** transient absorption kinetics of 350 and 370 nm, **(C)** schematic illustration of excited-state dynamics occurring in **Ir-dfpMepy-CN**. (The decay data in Figure 3B were fitted by a single-term exponential decay model: $y\left(t\right)=\;A_{0}*\text{exp}\left(-\frac{t}{\tau }\right)$ , where τ is the decay time and $A_{0}$ is a constant).

The excited-state dynamics of **Ir-dfpMepy-CN** are studied by the femtosecond transient absorption spectroscopy. The iridium complex in DCM solution was pumped by a flash laser at 320 nm, resulting a broad absorption ranging from 340 to 480 nm. Monitoring the kinetics of two absorption peaks at 350 and 370 nm gives two growth lifetimes of 0.9 and 1.2 picoseconds (ps), respectively, and this species has a long lifetime beyond 1,000 ps from fs-TA analysis, revealing the generation of triplet from singlet occurs in 1.2 ps. The non-decaying signal of the absorption spectra indicates the overlap of excited singlet and triplet states of the iridium complex, which adversely influences the relaxation between two energy states and leads to an almost constant transient absorbance (Hedley et al., 2008; Hedley et al., 2010). The schematic illustration of excited-state dynamics occurring in **Ir-dfpMepy-CN** is shown in Figure 3C. The relaxation process is proposed as a successive pathway from singlet to triplet and to S_0_ state with the relaxation time constant τ_1_ and τ_2_ (Tang et al., 2004). τ_1_ is obtained by transient absorption spectroscopy and τ_2_ is the phosphorescent decay time, which can be detected through previous kinetic study of PL measurement. **Ir-dfpMepy-CN**, as a novel [3+2+1] coordinated iridium complex, can achieve the transition lifetime of 0.9–1.2 ps and a phosphorescence lifetime of around 4.6 μs. The comprehensive understanding of the relaxation process of different energy states sheds light on the structural design of high-efficiency blue phosphors in future applications.

**FIGURE 4:**
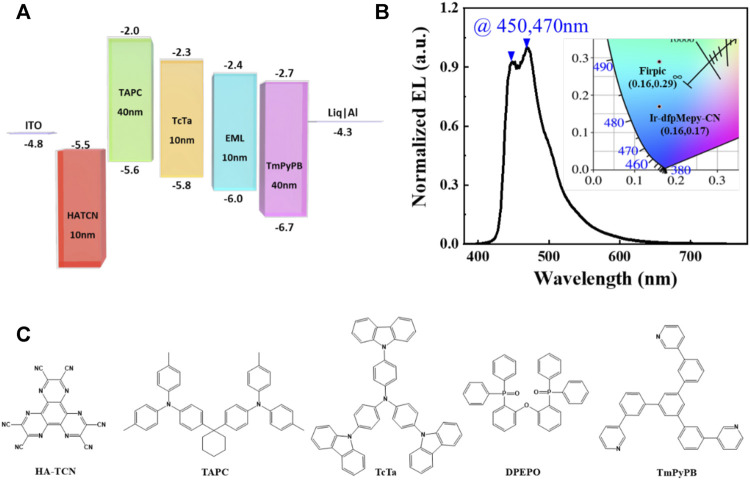
**(A)** The device structure and **(B)** electroluminescence (EL) spectra at 5 V of OLED fabricated with **Ir-dfpMepy-CN** as dopant (insert: CIE coordinate of **FIrpic** and **Ir-dfpMepy-CN**) **(C)** materials used for device fabrication.

The effectiveness of **Ir-dfpMepy-CN** is supported by the OLEDs device made from the novel [3+2+1] iridium complex as the dopant with physical vapor deposition technology. The detailed device structure is schematically shown in Figure 4A, including ITO as anode and aluminum as cathode. Matching the HOMO and LUMO energy levels of different functional materials, the active layers combined between the electrodes are HATCN (10 nm)/TAPC (40 nm)/TcTa (10 nm)/10 wt% **Ir-dfpMepy-CN** in DPEPO (10 nm)/TmpypB (40 nm)/Liq (2.5 nm)/Al (100 nm). The electroluminescence spectrum is depicted in Figure 4B. The molecular structure of the materials used in the device is shown in Figure 4C. Under applied voltage of 5 V, the device shows a stable luminescence with the two maximums at the peak wavelength of 450 and 470 nm separately, slightly different from the PL spectrum in Figure 2B. The electroluminescence shows a CIE chromaticity coordinate of (0.16, 0.17), reaching much deeper blue compared with that of the traditional FIrpic (CIE of (0.16, 0.29)). The current density, voltage, and Luminance (J-V-L) curve is depicted in Supplementary Figure S7. The device utilizing Ir-dfpMepy-CN as a blue dopant exhibits a turn-on voltage (Von) of 3.3 V and highest EQE of 4.2%, but a quick roll-off as the current density increases. The preliminary OLED device demonstrates that the newly designed and synthesized [3+2+1] conformation compound is amenable to thermally evaporated OLEDs. However, the performance of the device is far from satisfactory which needs further device optimization. Nevertheless, there is a possibility that the [3+2+1] phosphorescent materials may open a new window to explore for deep blue display applications.

## Conclusion

A novel [3+2+1] coordinated iridium (III) complex was designed and synthesized. The complex shows good stability due to the strong bonds formed by the center iridium atom and tridentate bis-NHC ligand. The designed [3+2+1] iridium (III) complex shows high photoluminescence peaks of 440 and 466 nm as both the bidentate (dfpMepy) and monodentate cyanide (-CN) ligand are strong electron-withdrawing groups, facilitating the stability of the HOMO levels. Notably, the complex shows a high PLQY of 84 ± 5% due to the rapid transition between singlet and triplet state with a time constant of 0.9–1.2 ps. The thermally evaporated device employed the [3+2+1] complex shows a CIE coordinate (0.16, 0.17), reaching deeper blue emission. This work provides a highly efficient blue cyclometalated iridium (III) complex configuration that will help develop more blue emitters and provide more dopant options for blue PhOLEDs.

## Data Availability

The original contributions presented in the study are included in the article/Supplementary Material, further inquiries can be directed to the corresponding author.
