# Supplementary material for: Highly Efficient Phosphorescent Blue-Emitting [3+2+1] Coordinated Iridium (III) Complex for OLED Application
Source: Front Chem. 2021 Oct 7;9:758357. doi: 10.3389/fchem.2021.758357 (PMC8529235; doi:10.3389/fchem.2021.758357)
Supplement: Supplementary file 1 [file DataSheet1.pdf]

Supplementary Material

The detailed procedure of measuring absolute quantum efficiency: 0.35 mg **Ir-dfpMepy-CN** was added into a 1ml glass bottle with 500 μl DCM. After the solution is evenly mixed, 100 μl mixed solution was transferred to a 5 ml volumetric flask DCM was then added into the 5 ml volumetric flask to get a 2×10^{-5} mol/L **Ir-dfpMepy-CN** solution. A cuvette with 2×10^{-5} mol/L **Ir-dfpMepy-CN** solution and reference cuvette are prepared for the quantum efficiency test by the absolute PL quantum yield spectrometer C11345 (Quantum-QY Hamamastu).

1 Supplementary Figures and Tables

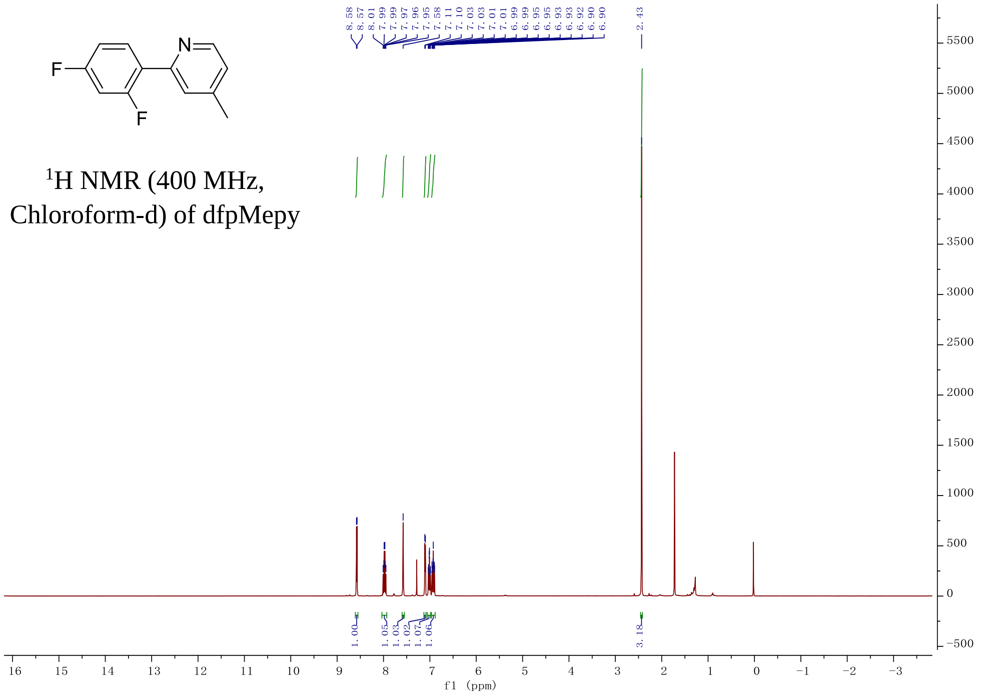

Supplementary figure 1. ^{1}H NMR of 2-(2,4-difluorophenyl)-4-methylpyridine(dfpMepy) in $CDCl_3$.

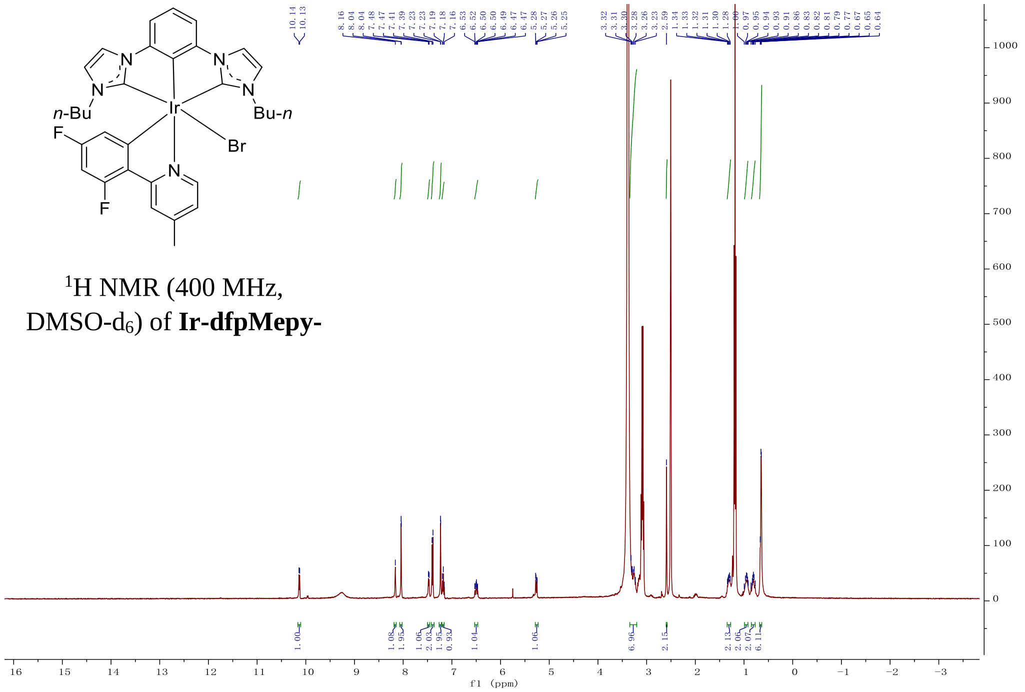

Supplementary figure 2. ^{1}H NMR of **Ir-dfpMepy-Br** in DMSO-d$_6$.

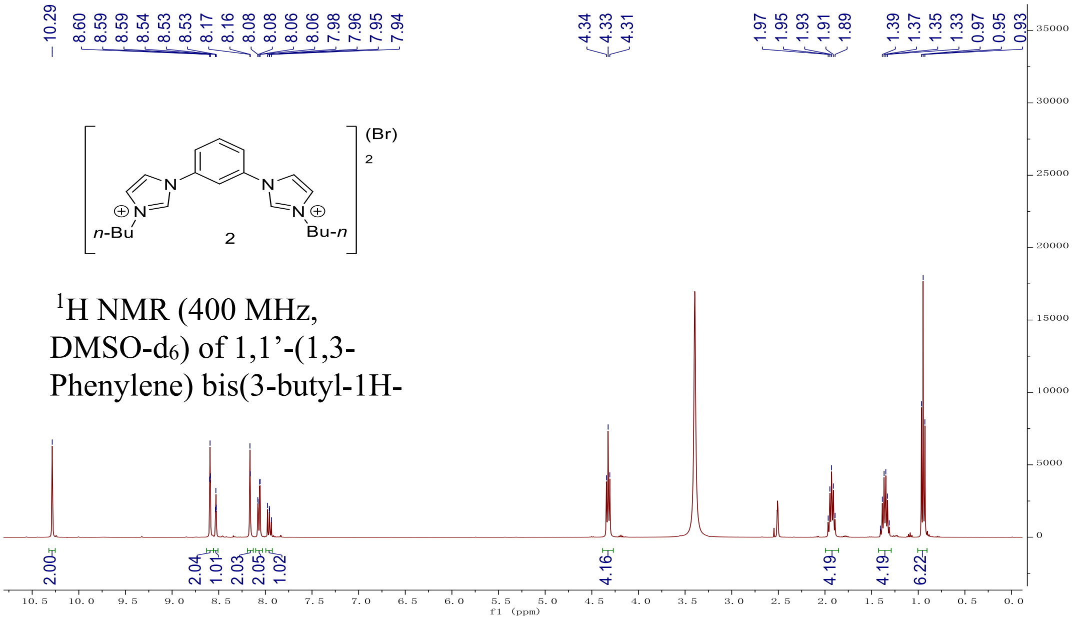

Supplementary figure 3. ^{1}H NMR of 1,1'-(1,3-Phenylene) bis(3-butyl-1H-imidazolium) Bromide in DMSO-d$_6$.

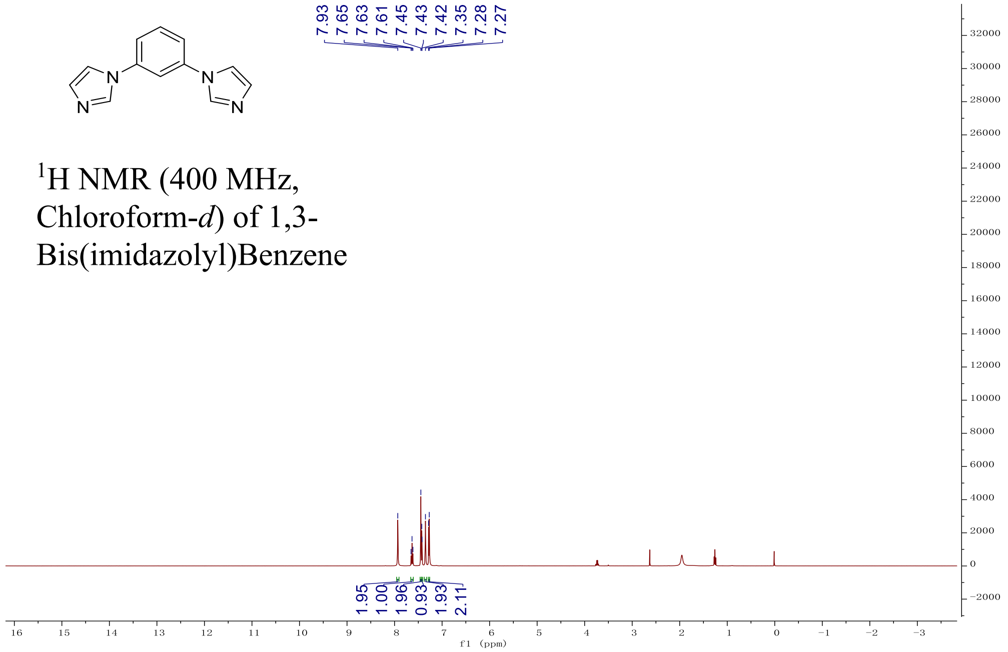

Supplementary figure 4. ^{1}H NMR of 1,3-Bis(imidazolyl)Benzene in $CDCl_3$.

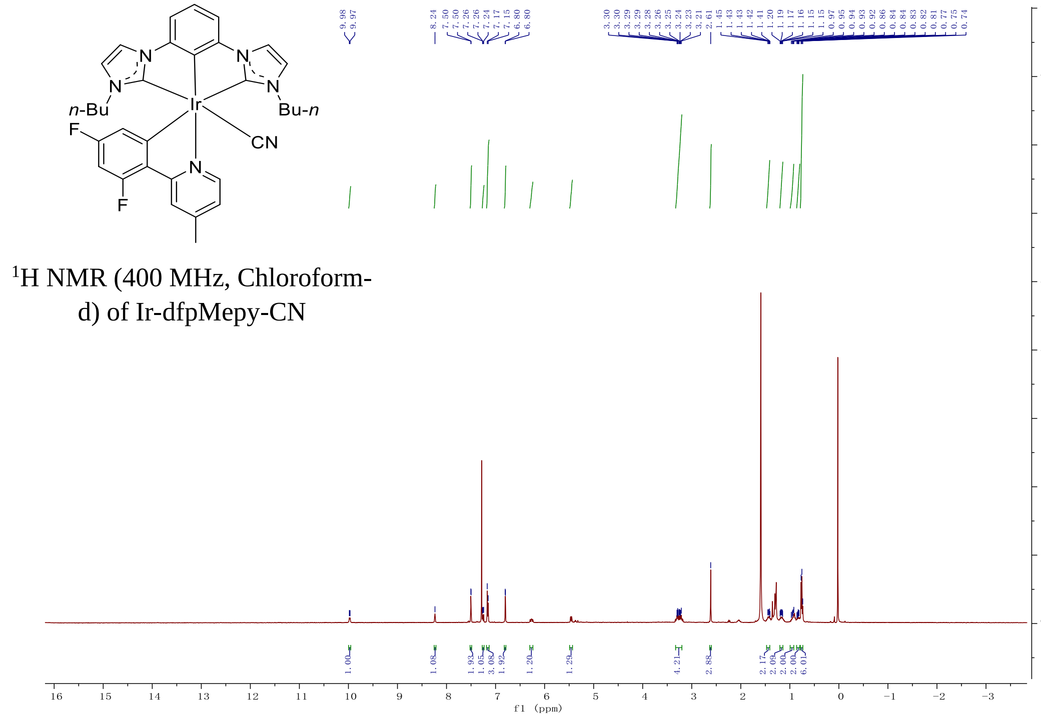

Supplementary figure 5. ^{1}H NMR of **Ir-dfpMepy-CN** in $CDCl_3$.

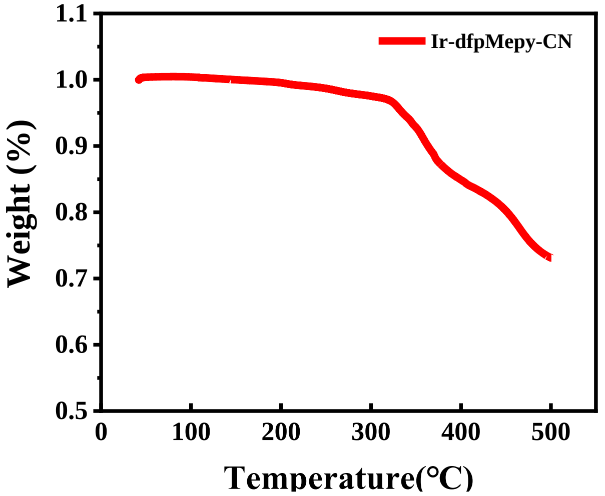

Supplementary figure 6. Thermogravimetric analysis of the **Ir-dfMeppy-CN.**

(a)

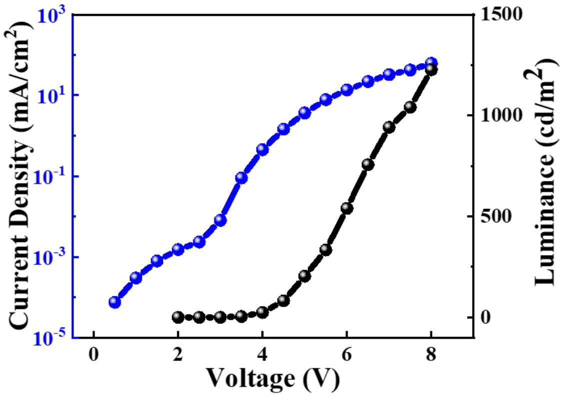

(b)

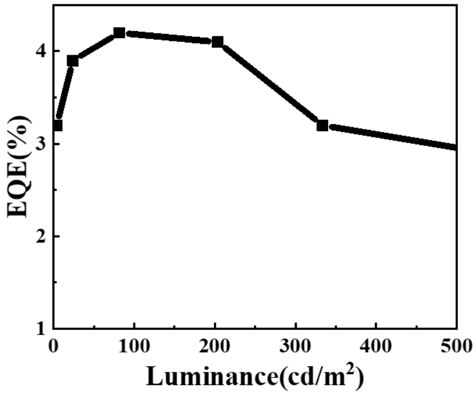

Supplementary figure 7. (a) J-Vand L-V curves and (b) External quantum efficiency of the device.

2 TD-DFT simulation results

Table S1 The calculated wavelength and charge transfer character of the optical transitions

	State	Energy(eV)	λ (nm)	Assignments
1	T1	2.8695	432	HOMO-3→LUMO 44.6% (LLCT & LC)<br>HOMO-2 → LUMO 12.5% (MLCT)<br>HOMO-1 → LUMO 21.8% (MLCT &LC)

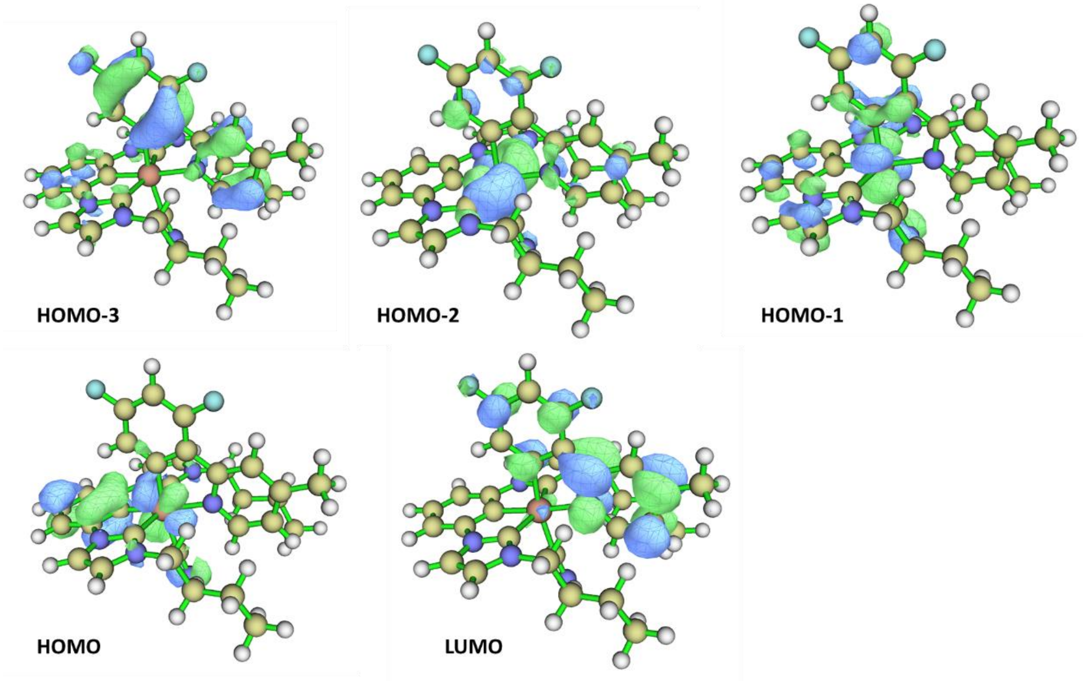

Supplementary figure 8. The frontier molecular orbitals calculated at T_1 geometries.
